# Regulation of Intestinal Immune Responses through TLR Activation: Implications for Pro- and Prebiotics

**DOI:** 10.3389/fimmu.2014.00060

**Published:** 2014-02-18

**Authors:** Sander de Kivit, Mary C. Tobin, Christopher B. Forsyth, Ali Keshavarzian, Alan L. Landay

**Affiliations:** ^1^Division of Digestive Diseases and Nutrition, Rush University Medical Center, Chicago, IL, USA; ^2^Department of Immunology/Microbiology, Rush University Medical Center, Chicago, IL, USA; ^3^Division of Pharmacology, Utrecht Institute for Pharmaceutical Sciences, Faculty of Science, Utrecht University, Utrecht, Netherlands

**Keywords:** toll-like receptors, intestinal epithelial cells, food allergy, microbiota, probiotics, prebiotics, circadian rhythm

## Abstract

The intestinal mucosa is constantly facing a high load of antigens including bacterial antigens derived from the microbiota and food. Despite this, the immune cells present in the gastrointestinal tract do not initiate a pro-inflammatory immune response. Toll-like receptors (TLRs) are pattern recognition receptors expressed by various cells in the gastrointestinal tract, including intestinal epithelial cells (IEC) and resident immune cells in the lamina propria. Many diseases, including chronic intestinal inflammation (e.g., inflammatory bowel disease), irritable bowel syndrome (IBS), allergic gastroenteritis (e.g., eosinophilic gastroenteritis and allergic IBS), and infections are nowadays associated with a deregulated microbiota. The microbiota may directly interact with TLR. In addition, differences in intestinal TLR expression in health and disease may suggest that TLRs play an essential role in disease pathogenesis and may be novel targets for therapy. TLR signaling in the gut is involved in either maintaining intestinal homeostasis or the induction of an inflammatory response. This mini review provides an overview of the current knowledge regarding the contribution of intestinal epithelial TLR signaling in both tolerance induction or promoting intestinal inflammation, with a focus on food allergy. We will also highlight a potential role of the microbiota in regulating gut immune responses, especially through TLR activation.

## The Mucosal Immune Response in the Intestine – An Overview

The mucosal tissue of the intestines contains the largest part of the immune system present in the human body, and is constantly exposed to many antigens, which are derived from amongst others food and micro-organisms including the commensal microbiota or invading pathogens. Approximately, 70% of the cells of the immune system are present in the gut and are continuously discriminating between harmless and pathogenic antigens. Nevertheless, the majority of oral foreign antigens do not result in inflammatory responses in healthy individuals. This phenomenon is known as oral tolerance. Local or systemic pathological inflammation may occur when oral tolerance toward some harmless luminal antigens is lost. This is seen for instance in food allergy, which is characterized by an inflammatory immune response toward generally harmless food-derived antigens.

Intestinal epithelial cells (IEC) provide a physical and chemical barrier between the intestinal lumen and the lamina propria. The expression of tight junction proteins by IEC, production of mucus by goblet cells and Paneth cell-derived antimicrobial peptides prevent translocation of luminal antigens and micro-organisms into the lamina propria (1, 2). Nevertheless, antigens are actively sampled into the gut-associated lymphoid tissue (GALT). Understanding of the GALT is essential to gain insight in both disease pathogenesis and to design new therapeutic strategies to prevent or cure inflammatory diseases of the intestine. As an antigen ends up in the lumen of the intestine, it is generally recognized by dendritic cells (DC) present in Peyer’s patches, after the antigen has been transported into the Peyer’s patch via specialized IEC known as M cells (3, 4). Antigen sampling also occurs via dendrites of DC that protrude between the IEC (5, 6). Upon antigen recognition, DC migrate toward the draining mesenteric lymph nodes (MLN) and activate T cells, which migrate back toward the intestinal lamina propria to carry out their effector functions (7).

Intestinal epithelial cells have been described to suppress DC activation as well and contribute to tolerance induction by secreting amongst others TSLP and TGF-β, and metabolize vitamin A into retinoic acid to induce the development of CD103^+^ DC (8–12). These CD103^+^ DC induce antigen-specific regulatory T cells (T_reg_) as well as the expression of the specific gut-homing molecules α4β7 integrin and CCR9 on T cells in the MLN (13). T_reg_ cells suppress adaptive immune responses through cell–cell contact dependent mechanisms or secretion of the anti-inflammatory cytokines IL-10 or TGF-β. Indeed, induction of T_reg_ cells results in abrogation of food hypersensitivity responses (14, 15). A higher frequency of allergen-specific T_reg_ cells is observed in children that have outgrown cow’s milk allergy and allergen-specific immunotherapy has been shown to induce T_reg_ cells (16, 17), implicating that the induction of T_reg_ cells is essential for mucosal tolerance.

## Regulation of Intestinal Immunity and Tolerance by TLRs Expressed by IEC

Toll-like receptors (TLRs) recognize a wide range of microbial fragments and therefore recognize both antigens derived from the microbiota as well as invading pathogens. TLRs are expressed by a variety of cells, including IEC. TLR2 can dimerize with TLR1 or TLR6 to recognize bacterial cell wall lipoproteins. LPS produced by Gram-negative bacteria is recognized by TLR4 in conjunction with CD14 and MD2, whereas unmethylated CpG motifs of bacterial DNA are recognized by TLR9. In addition, flagellin is recognized by TLR5, which is expressed at the basolateral membrane by IEC. TLR2, 4, and 5 are generally expressed at the cell membrane, whereas TLR9 is expressed intracellularly. However, in IEC, TLR9 has been reported to be expressed at the cell membrane as well (18, 19).

Under homeostatic conditions, IEC show low expression of TLR2 and TLR4 and are therefore unresponsive to TLR stimuli (20, 21). However, under inflammatory conditions, epithelial TLR expression is increased, which contributes to both inflammation as well as immune tolerance (19, 22, 23). Increased epithelial TLR2 and TLR4 expression is associated with inflammatory bowel disease (24). In contrast, apical TLR9 stimulation has been described to contribute to intestinal homeostasis (18). Interestingly, TLR activation of IEC appears to be important in regulating adaptive immune responses. Using an *in vitro* co-culture system, it was shown that TLR4 and basolateral TLR9 activation on IEC is important in driving an inflammatory response, whereas apical TLR9 activation supported the differentiation of an anti-inflammatory response (25). The underlying mechanisms by which TLR9 promotes tolerance are not well understood, but it has been described that apical but not basolateral TLR9 ligation on IEC prevents degradation of IκB-α, and therefore suppresses NF-κB-induced pro-inflammatory cytokine production by IEC (18). In addition, it has recently been indicated that apical TLR9 activation supports the expression and secretion of galectin-9, a soluble protein of the lectin family, which supports the differentiation of T_reg_ cells potentially by supporting the development of tolerogenic DC (26, 27). Though IEC are important in driving the development of tolerogenic CD103^+^ DC and suppress DC activation (8), it is not known whether TLR activation on IEC influences the generation of CD103^+^ DC. Recently, it has been shown that gut bacteria stimulate the recruitment of CD103^+^ DC into the epithelium potentially via TLR-dependent mechanisms in both IEC and hematopoietic cells (28). Altogether, TLR stimulation in the intestinal epithelium plays an important role in regulating mucosal immune responses in the intestine.

In addition to regulating intestinal immunity, TLR activation on IEC is also known to modulate the expression of tight junction proteins. In many inflammatory disorders, including food allergy, epithelial tight junctions are impaired and increased bacterial translocation occurs (29). This increased bacterial translocation into the lamina propria may sustain the inflammatory response. In particular, epithelial TLR2 activation has been described to protect against barrier disruption by enhancing zonula occludens (ZO)-1 expression in IEC in a protein kinase C-dependent manner (30). In contrast, activation of TLR4 increases intestinal permeability and results in enhances bacterial translocation (31). NF-κB signaling as a result of TLR4 activation by LPS appears to play a major role in LPS-mediated barrier disruption (32, 33). Similarly, apical *Campylobacter jejuni* infection of T84 cell monolayers results in a rapid decrease in the transepithelial resistance of the monolayer involving NF-κB signaling (34). Activation of TLR9 apically on IEC prevents TLR4-induced gut leakiness and infection of IEC monolayers with *Campylobacter jejuni* disrupts the intestinal epithelial barrier function by reducing TLR9 expression at the surface membrane of IEC (33). In this similar study, the authors also indicate an increase in the intestinal barrier function upon apical, but not basolateral TLR9 stimulation with a synthetic CpG DNA (35). Preliminary data from our group also report a potential protective effect of apical TLR9 activation in T84 cell monolayers co-cultured with CD3/28-activated PBMC. Hence, paracellular transport of antigens as well as bacterial translocation under pathological conditions may be affected by TLR activation on IEC.

With respect to food and environmental allergens, the contribution of TLR activation on IEC is not well studied. Recently, TLR4 activation by wheat α-amylase trypsin inhibitors, a recognized plant-derived allergen (36), has been described to drive intestinal inflammation (37). The percentage of α-amylase trypsin inhibitors is markedly higher in genetically modified grain seeds that are more resistant to infection than traditional seeds (38–40), which might explain why a wheat-free diet could be beneficial in a wide range of inflammatory and allergic disorders. Similarly, the house dust mite allergen Der p 2 as well as the major cat allergen Fel d 1 enhance signaling through TLR2 and TLR4 (41). Although these studies were carried out on innate immune cells, this does not exclude that these allergens may interact with TLR expressed by IEC as well. Especially, since TLR activation on IEC affects the mucosal barrier function and potentially shapes mucosal immune responses in the intestine, interactions of allergens with TLR expressed by IEC may facilitate their entry into the gut mucosa and sustain the allergic inflammatory response. Interestingly, treatment with CpG oligodeoxynucleotides improved the intestinal barrier function and increased the percentage of T_reg_ cells in the spleen and MLN (42). Since epithelial TLR may interact with the gut microbiota and luminal antigens, further understanding of the role of epithelial TLR activation in food allergy is necessary.

## Interactions between the Microbiota and TLRs

The microbiota is the largest source of microbial stimulation in the gut. Furthermore, the microbiota is necessary for development of the intestinal immune system (43). The “hygiene hypothesis,” currently the most popular theory of deregulation of the microbiota, theorizes that specific microbial stimulation is necessary for gut health. Originally, it states that microbial stimulation polarizes the immune response toward T_h_1, while lack of microbial stimulation maintains a T_h_2 polarized immune response, which is characteristic for atopy (44). Recently, a specific microbiota signature was linked to oral allergic sensitization in mice exhibiting a gain-of-function mutation in the IL-4 receptor α chain, which rendered these animals more prone to developing food allergy. This microbiota signature was characterized by a reduction in *Firmicutes* spp. and increase in *Proteobacteria* spp. (45). Another example that indicates the importance of the gut microbiota composition in the development of food allergy is a recent study showing that colonization of germ-free mice with the fecal microbiota of a healthy infant rich in *Bifidobacterium* spp. and *Bacteroides* spp. protected against the development of cow’s milk allergy following sensitization to β-lactoglobulin (46). This was associated with lower T cell reactivity toward the allergen, an increase in Foxp3^+^ T_reg_ and lower bacterial translocation into the lamina propria. *Bifidobacterium breve* potentially activates CD103^+^ intestinal DC to produce IL-10 and IL-27 in a TLR2-dependent fashion to induce IL-10-producing T_r_1 cells (47), whereas colonization of germ-free mice with *Bacteroides fragilis* restores the T_h_1/T_h_2 balance and prevents intestinal inflammation through induction of IL-10 producing CD4^+^ T cells. This was dependent on recognition of *B. fragilis* polysaccharide A by gut DC (48, 49).

Disturbances in the commensal bacterial composition in the gut, reflected by increased colonization with *Escherichia coli* or *Clostridium difficile*, is associated with an increased risk in the development of allergic disease and IBD in humans (50, 51). The fecal microbiota of allergic infants shows a higher prevalence of *Clostridium* spp. and *Staphylococcus aureus*. In parallel, lower levels of *Bifidobacteria, Enterococci*, and *Bacteroides* were found in the stool of allergic infants compared to healthy individuals (52, 53). Bacterial colonization early in life has been shown to affect cytokine production by T helper cell subsets, implicating that dysbiosis at an early age may increase the risk of developing food allergy (54). Likewise, infants that have developed eczema by the age of 12 months show a lower diversity in the gut microbiota during the early postnatal period (55). Thus, it appears that low abundance of *Bifidobacteria, Enterococci*, and *Bacteroides* and a higher abundance of *Clostridium* spp. and *Staphylococcus* are associated with loss of tolerance and an exaggerated allergic response toward food-derived antigens. However, it was recently shown that *Clostridium butyricum* can induce IL-10 producing macrophages in the gut in a TLR2-dependent manner and suppresses TLR4 expression by colonic IEC (56, 57). Hence, host–microbiome interactions not only promote a normal T_h_1/T_h_2 balance, but support the development of T_reg_ responses as well. Whether changes in microbiota composition are a factor to promote an allergic response to food or are a consequence of food allergy remains to be studied.

It is important to note that not only changes in the microbiota are present in individuals with food allergy, but the response of immune cells toward the microbiota has also been described to be different. The so-called beneficial bacteria are not necessarily associated with anti-inflammatory responses in allergic patients. For example, although an increased prevalence of *Bifidobacteria* is rendered as beneficial, specific *Bifidobacterium* strains isolated from the feces of allergic infants were shown to induce increased production of the pro-inflammatory cytokines IL-1β, IL-6, and TNF-α (58). This is supported by the observation that the allergic infants showed an increased IL-6 and TNF-α response toward TLR2, TLR4, and TLR5 stimuli (59).

Using *in vitro* models it was shown that IEC play an important role in discrimination between different bacterial strains at the apical membrane (60, 61). In addition, commensal bacteria have the capacity to enhance TLR expression by IEC (62–66). This suggests that TLR responses toward microflora constituents may be important. However, not all bacterial strains are equally effective in suppressing food allergy. This is reflected by the selective capacity of bacterial strains to induce Foxp3^+^ T_reg_ cells in a murine model for OVA-induced asthma and OVA-induced food hypersensitivity (67). Similarly, only specific *Lactobacillus* strains attenuate T_h_2 responses by inducing CD103^+^ tolerogenic DC (68). Both *Lactobacillus* and *Bifidobacterium* strains have been shown to induce T_reg_ type immune responses, thereby suppressing allergy (47, 69–72). Recently, it has been shown that the bacterial DNA from *Lactobacillus* spp. or probiotics contain a higher frequency of immunoregulatory CpG motifs – potentially stimulating TLR9 – when compared to pathogenic bacteria like *E. coli*, which is important for T_reg_ conversion in the intestinal mucosa (73). Exposure of IEC to DNA derived from *E. coli* or *S. dublin* induces high IL-8 production by IEC (19, 74), whereas DNA from *Lactobacillus rhamnosus GG* prevents NF-κB-induced IL-8 production by IEC (66). Similarly, apical exposure of IEC to genomic DNA from *B. breve* M-16V was found to enhance IFN-γ and IL-10 secretion by PBMC in an HT-29/PBMC co-culture model (26). In line with this study, it was shown that DC cultured in the conditioned medium of IEC apically exposed to *S. Dublin* DNA, but not from *B. breve*, produced increased amounts of pro-inflammatory cytokines (75). This suggests that not all probiotic bacterial strains are potentially effective in treating allergic diseases. Selection of probiotic bacterial strains should possibly be based on their richness in CpG motifs, targeting TLR9, and bacterial strains high in these motifs may be considered for clinical trials.

## Prebiotics Shape the Intestinal Microbiota

Breast feeding also affects the microbiota composition by increasing the amount of *Bifidobacteria* as shown by higher fecal *Bifidobacteria* counts (76). Human milk contains a high amount of non-digestible oligosaccharides with over 1000 different oligosaccharide structures and it has been shown that human milk, as well as specific dietary fibers like chicory-derived inulin and lactose-derived short-chain galacto-oligosaccharides (scGOS), selectively support the growth of *Lactobacillus* and *Bifidobacterium* strains (77). Therefore, these oligosaccharides have prebiotic effects in the intestine. Based on the basic structure and size of neutral non-digestible oligosaccharides present in human milk, a specific prebiotic mixture consisting of scGOS and long-chain fructo-oligosaccharides (lcFOS) in a 9:1 ratio has been developed. Oral supplementation of scGOS/lcFOS has been shown to reduce allergic symptoms in mice and humans (78–80). Especially dietary supplementation with a combination of scGOS/lcFOS and *B. breve* M-16V (GF/*Bb*) is effective in reducing allergic symptoms (81, 82). In a colitis model in rats, inulin, and FOS reduced colitis, which was associated with increased *Bifidobacterium* species and reduced *Enterobacteriaceae* and *C. difficile* in the feces (83). The underlying mechanisms are not known. However, exposure of IEC to GF/*Bb* may result in the generation of tolerogenic DC and consequently T_reg_ polarization in the GALT. In addition to supporting T_reg_ conversion, stimulation of the growth of *Lactobacillus* and *Bifidobacterium* strains may also improve the intestinal barrier function in a TLR2 and potentially TLR9 dependent manner (84, 85).

## Circadian Clock and TLR

Although the type of microbiota composition is a critical factor for the state of TLR activation in the gut of patients with allergic disorders, other environmental factors can also influence TLR activation. It has recently been shown that the expression of TLRs is under regulation of the circadian clock. This implicates that the expression of TLRs is not temporally fixed in a 24-h day and night cycle. Recently, the expression of TLR9 as well as other TLRs were shown to be regulated by the circadian clock (86, 87). Interestingly, the severity of TLR9-mediated induction of sepsis is associated with the time-dependent expression of TLR9 (86). Moreover, further studies have indicated that the interaction between the microbiota and TLRs expressed by the gut epithelium is dependent on the circadian rhythm as well (88). Besides the observation that the expression of TLRs is under circadian control, cytokine production by macrophages and CD4^+^ T cells, the suppressor function of Foxp3^+^ T_reg_ cells, leukocyte trafficking, and antibody production also show a circadian pattern (89–97). Furthermore, it was recently shown that the circadian clock is critical for regulation of intestinal permeability as well, as disruption of the circadian rhythm led to increased microbial translocation and disruption of the epithelial tight junctions (98). Hence, interactions between the microbiota and the intestinal mucosal immune system may not only be dependent on the type of bacterial species present in the microbiome, but are also temporally regulated, which may contribute to regulation of immune responses in the intestine. These data may explain why many allergic reactions like asthma attacks occur in the early morning (99, 100). Recently, it was shown that the expression of the FcεRI by mast cells and IgE-mediated mast cell degranulation is temporally regulated by the circadian clock (101, 102). Also, it might, at least partially, explain the rapid rise of incidence of (food) allergies in western societies where disruption of normal circadian patterns and stress is a consequence of modern day society (103).

## Implications for the Use of Pro- and Prebiotics

**Figure 1:**
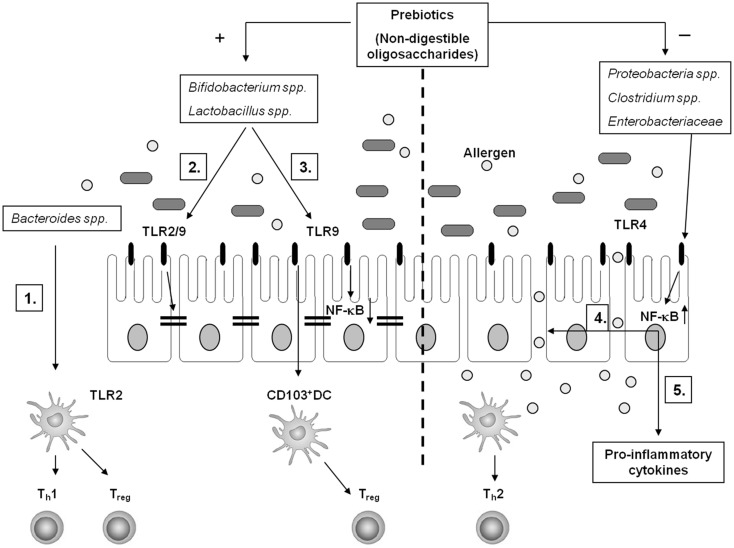
**Schematic overview of potential interactions between the gut microbiota and the intestinal mucosal immune system**. A healthy gut microbiota composition is high in the frequency of *Bacteroides* spp., *Lactobacillus* spp., and *Bifidobacterium* spp. (1) In particular, *Bacteroides fragilis* supports T_h_1 and T_reg_ polarization in a TLR2-dependent manner through recognition of polysaccharide A by gut DC. Genomic DNA of *Bifidobacterium* spp. and *Lactobacillus* spp. – rich in unmethylated CpG motifs – potentially interact with TLR2 and/or TLR9 to enhance the intestinal epithelial barrier function (2) and to support T_reg_ conversion via CD103^+^ DC (3). Furthermore, apical TLR9 activation by IEC suppresses NF-κB activation (3). In food allergy, the microbiota composition shifts toward a higher frequency in *Proteobacteria* spp., *Clostridium* spp., and *Enterobacteriaceae*. This may favor TLR4 mediated barrier disruption facilitating allergen translocation in the gut mucosa (4) and pro-inflammatory cytokine production (5) in a NF-κB-dependent fashion, sustaining an allergic inflammation. Specific non-digestible oligosaccharides (prebiotics) support the growth of *Bifidobacterium* spp. and *Lactobacillus* spp. and suppresses the growth of *Clostridium* spp. and *Enterobacteriaceae*, which may contribute to induction of tolerance toward allergens in the intestines.

There is still controversy about the effectiveness of probiotic and prebiotic treatment in food allergy (104). However, given the data that alteration of the gut microbiota influences mucosal immune responses in the gut indicates that treatment using specific probiotic bacterial strains as well as prebiotics may be useful in treatment for food allergy (Figure 1). Selection of the right bacterial strains appears key to the effect of treatment using probiotics. Especially, characterization of specific probiotics based on CpG rich motifs in the DNA may improve the selection of potential beneficial strains. Hence, studies aimed at the interaction between probiotic bacteria and epithelial expressed TLRs may be warranted. In addition, timing of treatment may play an essential factor in the effectiveness of treatment using pro- and prebiotics as expression of TLRs and immune cell functions appears to be regulated by the circadian clock. In conclusion, more studies are necessary focusing on interaction between the gut epithelium and gut bacteria, both in terms of selecting potential beneficial bacterial strains as well as appropriate timing of intervention.

## Author Contributions

Sander de Kivit wrote the manuscript; Mary C. Tobin, Christopher B. Forsyth carefully reviewed the manuscript; Ali Keshavarzian and Alan L. Landay reviewed the manuscript and provided overall supervision.

## Conflict of Interest Statement

The authors declare that the research was conducted in the absence of any commercial or financial relationships that could be construed as a potential conflict of interest.
